# Educational Level, but Not Income or Area Deprivation, is Related to Macrovascular Disease: Results From Two Population-Based Cohorts in Germany

**DOI:** 10.3389/ijph.2021.633909

**Published:** 2021-05-10

**Authors:** Violetta Ptushkina, Esther Seidel-Jacobs, Werner Maier, Sabine Schipf, Henry Völzke, Marcello Ricardo Paulista Markus, Matthias Nauck, Christa Meisinger, Annette Peters, Christian Herder, Lars Schwettmann, Marcus Dörr, Stephan B. Felix, Michael Roden, Wolfgang Rathmann

**Affiliations:** ^1^ Institute for Biometrics and Epidemiology, German Diabetes Center (DDZ), Leibniz Center for Diabetes, Research at Heinrich-Heine-University, Düsseldorf, Germany; ^2^ German Center for Diabetes Research (DZD e.V.), Partner Düsseldorf, München-Neuherberg, Germany; ^3^ Institute of Health Economics and Health Care Management, Helmholtz Zentrum München, German Research Center for Environmental Health (GmbH), München-Neuherberg, Germany; ^4^ Institute for Community Medicine, University Medicine Greifswald, Greifswald, Germany; ^5^ German Center for Cardiovascular Research (DZHK), Partner Site Greifswald, Greifswald, Germany; ^6^ Department of Internal Medicine B, University Medicine Greifswald, Greifswald, Germany; ^7^ Institute of Clinical Chemistry and Laboratory Medicine, University Medicine Greifswald, Greifswald, Germany; ^8^ Independent Research Group Clinical Epidemiology, Helmholtz Zentrum München, German Research Center for Environmental Health, München-Neuherberg, Germany; ^9^ Chair of Epidemiology, University of Augsburg at University Hospital of Augsburg, Augsburg, Germany; ^10^ Institute of Epidemiology, Helmholtz Zentrum München, German Research Center for Environmental Health (GmbH), München-Neuherberg, Germany; ^11^ Institute for Clinical Diabetology, German Diabetes Center (DDZ), Leibniz Center for Diabetes Research at Heinrich-Heine-University, Düsseldorf, Germany; ^12^ Department of Endocrinology and Diabetology, Medical Faculty and University Hospital Düsseldorf, Heinrich-Heine-University, Düsseldorf, Germany; ^13^ Department of Economics, Martin Luther University Halle-Wittenberg, Halle (Saale), Germany

**Keywords:** macrovascular disease, education, income, area deprivation, population-based studies

## Abstract

**Objectives:** An inverse relationship between education and cardiovascular risk has been described, however, the combined association of education, income, and neighborhood socioeconomic status with macrovascular disease is less clear. The aim of this study was to evaluate the association of educational level, equivalent household income and area deprivation with macrovascular disease in Germany.

**Methods:** Cross-sectional data from two representative German population-based studies, SHIP-TREND (*n* = 3,731) and KORA-F4 (*n* = 2,870), were analyzed. Multivariable logistic regression models were applied to estimate odds ratios and 95% confidence intervals for the association between socioeconomic determinants and macrovascular disease (defined as self-reported myocardial infarction or stroke).

**Results:** The study showed a higher odds of prevalent macrovascular disease in men with low and middle educational level compared to men with high education. Area deprivation and equivalent income were not related to myocardial infarction or stroke in any of the models.

**Conclusion:** Educational level, but not income or area deprivation, is significantly related to the macrovascular disease in men. Effective prevention of macrovascular disease should therefore start with investing in individual education.

## Introduction

Macrovascular diseases, including myocardial infarction and stroke, belong to the global leading causes of morbidity [1] and remain the most common cause of death, accounting for 17.9 million deaths worldwide in 2016 [2]. Prevention of macrovascular disease requires the reduction of associated lifestyle-related risk factors like physical inactivity, smoking, risky alcohol consumption or low dietary fruit and vegetable intake [3]. There is a growing evidence that in addition to these well-known risk factors, socioeconomic factors like educational level [4] or household income [5] at the individual level, as well as area deprivation [6] at the neighborhood level, are also associated with macrovascular disease. Moreover, as demonstrated by Geyer at al., these socioeconomic determinants are each independently linked to health outcomes, since they intervene into various causal mechanism [7].

In Germany, regional differences in the prevalence of macrovascular disease exist [8]. A previous analysis provided evidence for a higher prevalence of macrovascular morbidity in the northeastern than in the southern region of Germany [8]. However, traditional risk factors, such as male sex, higher age, smoking and overweight were not able to fully explain this regional variation [8]. Studies investigating the impact of individual and area levels of socioeconomic factors on macrovascular complications may provide further explanations for this observation. This could be used to develop efficient strategies through better focusing on high-risk groups. Most of previous studies demonstrated an association between low educational level, low income, and high area deprivation with an increased risk for macrovascular complications [4, 6, 9]. Nevertheless, a study from France [10] reported no impact of income and area deprivation on the incidence of coronary heart disease.

In Germany, such studies are rare, and no study has assessed the associations between myocardial infarction and stroke including three different socioeconomic determinants (education, income and area deprivation) simultaneously.

Some previous studies on the associations between macrovascular outcomes and socioeconomic factors came to inconsistent results. Therefore, we aimed to study the impact of education, income and area deprivation on macrovascular disease in Germany, using these factors separately and together in regression models.

## Methods

### Study Populations

For the present analysis, data from two different cohort studies: SHIP-TREND (Study of Health in Pomerania) and KORA-F4 (Cooperative Health Research in the Region Augsburg) were used.

#### SHIP-TREND Study (Northeast of Germany)

The SHIP-TREND study region is located in the Northeast of Germany. A stratified random sample of adults aged 20–79 years with German nationality was drawn from a central population registry of Western Pomerania (212,157 inhabitants) with aiming to assess prevalence and incidence of common risk factors, subclinical disorders and clinical diseases in the German population. A two-stage cluster sampling method was used which followed the World Health Organization (WHO) Multinational Monitoring of Trends and Determinants in Cardiovascular disease (MONICA) Project in Germany. Of all persons invited, 4,420 (50.1%) individuals took part in the examinations between 2008 and 2012. All participants provided written informed consent and the medical ethics committee of the University of Greifswald approved the study protocol. Further information on the study design has been described in detail elsewhere [11].

#### KORA-F4 Study (South of Germany)

The KORA-F4 study (2006–2008) is the 7 years follow-up of the KORA-S4 study (1999–2001), a population-based health survey, which was conducted in the city of Augsburg and the two surrounding counties (about 600,000 inhabitants). The survey sampling method of the former WHO MONICA project was also used. Of the 4,261 participants with German nationality aged 25–74 years in S4, 3,080 took part in the F4 study (72.3%). The loss of participants from S4 to F4 occurred due to deaths (*n* = 176), demands for the deletion of data (*n* = 12), or because participants were completely lost to the follow-up (206), could not be contacted (*n* = 174), were unable to come (*n* = 218) or refused to participate (*n* = 395) [12]. All study participants gave written informed consent to the study. The study design was approved by the ethics committee of the Bavarian Medical Association. The study design, sampling method and data collection have been previously published [13].

### Variables

In both studies, information on demographic and socioeconomic variables, lifestyle habits and medical history were collected by trained and certified staff during standardized personal interviews [11, 13].

#### Key Measurements

Macrovascular disease was defined as composite endpoint of self-reported previous myocardial infarction or stroke. The presence of these diseases was obtained by the questions: “Have you ever had a myocardial infarction diagnosed by a physician?” and “Have you ever had a stroke diagnosed by a physician?”

#### Measures of Individual Socioeconomic Status

• Educational level was classified in two groups: low/middle (less than university qualification) or high level (university qualification). According to the German school system, low educational level includes participants with up to 9 years of schooling, middle educational level is equivalent to 10 years of schooling and high educational level to 12 or 13 years of schooling, which is the general qualification for university entrance [14].
• Equivalent income was calculated according to the Luxembourg Income Study (income/household size^0.36^) [14, 15]. First, the median income was calculated for each study separately. Second, four income groups were differentiated: low equivalent income (<60% of the study-specific median income), lower middle equivalent income (≥60% up to 100%), upper middle equivalent income (>100% up to ≤150%) and high equivalent income (>150%). Finally, the results were pooled to receive a standardized equivalent income with simultaneous consideration of income differences between the two regions.

Area deprivation was assessed by the German Index of Multiple Deprivation (GIMD), which has been established based on methods used in the United Kingdom [16]. The GIMD includes seven different domains of deprivation (income, employment, education, municipal revenue, social capital, environment and security). The index is divided in quintiles, in which quintile 1 includes least deprived and quintile 5 the most deprived municipalities. Due to the small number of cases in quintiles 1 to 4 in SHIP-TREND and in quintile 5 in KORA-F4, in the present analysis the index was dichotomized contrasting low/middle deprivation (quintiles 1–3) and high deprivation (quintiles 4 and 5). More details on the GIMD have been previously published [14].

#### Covariates

Participants were classified as current smokers if they smoked at least one cigarette per day regularly, as ex-smokers if they had quit smoking more than 12 months ago and as non-smokers if they had either never smoked or less than one cigarette per day. Known diabetes (type 1, type 2 and other forms) was defined using self-reported diagnosis. In KORA-F4, this information was validated by a physician [17]. Anthropometric measurements were taken after removing shoes and heavy clothing. Body mass index (BMI) was calculated as weight [kg] divided by height^2^ [m^2^]. Blood pressure measurements were taken at the right arm after a rest period of at least 5 min in a sitting position and repeated three times at an interval of 3 min. The final value was calculated as a mean of the second and third measurement. In SHIP-TREND, total serum cholesterol, LDL-, HDL-cholesterol and triglycerides were measured using the Dimension Vista 500 analytical system (Siemens AG, Erlangen, Germany). In KORA-F4, serum LDL- and HDL cholesterol were accessed by an enzymatic method (CHOD-PAP, LDL Flex and AHDL Flex, Dade Behring) and serum triglycerides were quantified by the enzymatic GPO-PAP method (TGL Flex, Dade Behring).

### Statistical Analyses

Normally distributed continuous variables are presented as means and standard deviations (SD), variables with skewed distribution as medians and interquartile ranges (25th and 75th percentiles). Categorical variables are reported with numbers and percentages. Differences between groups were assessed using *t*-tests for continuous variables and chi-square tests for categorical variables. Multilevel modeling methods would have been the preferred approach to link individual-level data to the data of neighborhood deprivation. However, we were not able to use multilevel models because of privacy regulations in SHIP-TREND study. Therefore, multivariate logistic regression models were performed using macrovascular disease as the dependent variable and individual educational level, individual equivalent income and area deprivation as the independent variables of interest.

Odds ratios (OR) and 95% confidence intervals (95% CI) were calculated by fitting of following models:

(0) Crude models (adjusted for cohort, sex, age, educational level, equivalent income and GIMD, respectively);
  (1–5) Adjusted for cohort, sex, age and
  (1) educational level,
  (2) equivalent income,
  (3) GIMD,
  (4) educational level and GIMD,
  (5) educational level and equivalent income;

(6–10) Adjusted for cohort, sex, age, further potential confounders (diabetes, BMI, blood pressure, LDL-cholesterol, triglycerides and smoking) and

(6) educational level,
(7) equivalent income,
(8) GIMD,
(9) educational level and GIMD,
(10) educational level and equivalent income.

Finally, interaction terms were added to the multivariate models 6 (GIMD and educational level) and 7 (GIMD and equivalent income) to assess possible interaction between area deprivation and individual socioeconomic characteristics on macrovascular disease.

All models were fitted separately for men, women and total population. A complete case analysis was conducted. A two-sided alpha level of 0.05 was chosen as criterion for statistical significance. All analyses were performed using SAS 9.4 (SAS Institute Inc., Cary, NC, United States).

## Results

### Participants’ Characteristics

**FIGURE 1:**
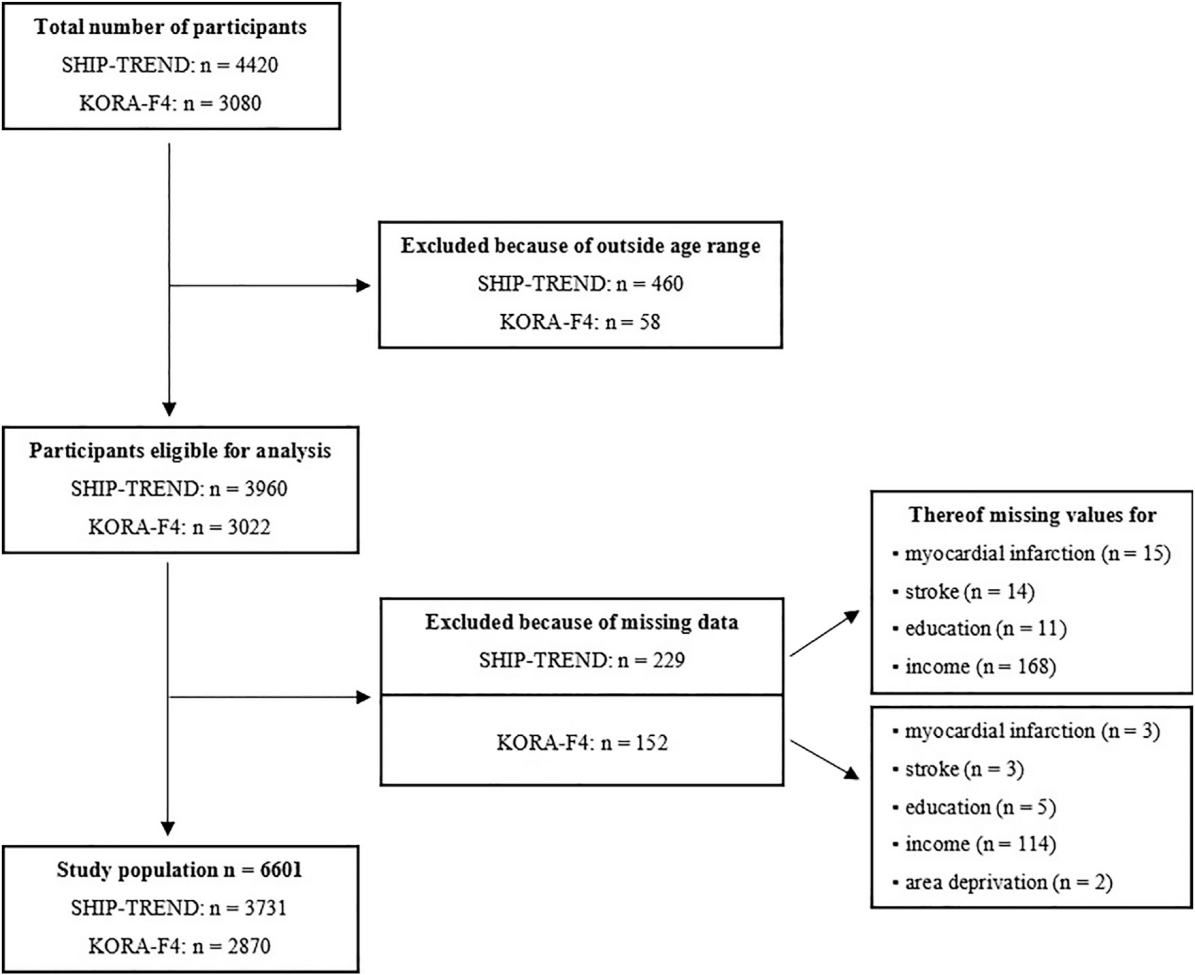
Flowchart showing the numbers of participants of Study of Health in Pomerania (2008–2012) and Cooperative Health Research in the Region Augsburg (2006–2008) at each stage of selection, Germany.

After excluding all participants without complete study data, 3,731 (94.2%) SHIP-TREND and 2,870 (95.0%) KORA-F4 participants aged 30–79 years were included (Figure 1 “Flowchart showing the numbers of participants at each stage of selection”). The baseline characteristics of the participants stratified by cohort and area deprivation are presented in Table 1. A total of 3,332 (89%) participants in SHIP-TREND and 1,208 (42%) participants in KORA-F4 lived in highly deprived areas. In both studies, these participants were older, had higher triglycerides values and a higher prevalence of current smoking. Additionally, in SHIP-TREND, people who lived in more deprived areas had a higher BMI, a higher prevalence of diabetes, higher systolic blood pressure, had lower equivalent income and were more often low or middle educated. In KORA-F4, differences for education and income between areas were less pronounced. Overall, SHIP-TREND participants were younger, had a slightly higher BMI, blood pressure and triglycerides values, but somewhat lower LDL-cholesterol than KORA-F4 participants. Current smoking was reported more often in SHIP-TREND, whereas ex-smoking was observed more frequently in KORA-F4. The prevalence of diabetes and stroke was significantly higher in SHIP-TREND than in KORA-F4. Sex distribution as well as frequency of low/middle educational level were similar in both cohorts.

**TABLE 1:** Baseline characteristics of participant of study of health in Pomerania (2008–2012) and cooperative health research in the region Augsburg (2006–2008) by cohort and area deprivation, Germany.

	SHIP-TREND				KORA-F4				*p*-value^b^
	Total	Low/middle deprivation	High deprivation	*p*-value^a^	Total	Low/middle deprivation	High deprivation	*p*-value^a^	
N (%)	3731	399 (11)	3332 (89)		2870	1662 (58)	1208 (42)		
Age (years)	53.9 (13.3)	51.4 (12.5)	54.2 (13.4)	**<0.001**	55.7 (12.9)	55.0 (12.9)	56.5 (12.8)	**0.003**	**<0.001**
Female sex	1797 (51.8)	218 (54.6)	1716 (51.5)	0.236	1463 (51.0)	856 (51.5)	607 (50.3)	0.506	0.488
Body mass index (kg/m^2^)	28.4 (5.2)	27.7 (5.0)	28.5 (5.2)	**0.004**	27.6 (4.8)	27.7 (4.8)	27.6 (4.8)	0.554	**<0.001**
Diabetes	398 (10.7)	22 (5.5)	376 (11.3)	**0.001**	233 (8.1)	131 (7.9)	102 (8.4)	0.587	**0.001**
Systolic blood pressure (mmHg)	128.6 (18.7)	126.0 (18.3)	128.9 (18.7)	**0.004**	122.2 (18.6)	121.9 (18.6)	122.7 (18.5)	0.248	**<0.001**
Diastolic blood pressure (mmHg)	77.9 (10.2)	77.1 (9.8)	78.0 (10.3)	0.071	75.2 (10.0)	75.2 (10.0)	75.3 (9.9)	0.876	**<0.001**
Total cholesterol (mmol/L)	5.5 (1.1)	5.5 (1.1)	5.5 (1.1)	0.345	5.6 (1.0)	5.6 (1.0)	5.6 (1.0)	0.689	0.057
HDL-cholesterol (mmol/L)	1.4 (0.4)	1.5 (0.4)	1.4 (0.4)	0.083	1.4 (0.4)	1.4 (0.4)	1.4 (0.4)	0.840	0.411
LDL-cholesterol (mmol/L)	3.4 (1.0)	3.4 (0.9)	3.4 (1.0)	0.349	3.5 (0.9)	3.5 (0.9)	3.5 (0.9)	0.589	**<0.001**
Triglycerides (mmol/L)	1.4 (1.0–2.1)	1.3 (0.9–1.8)	1.4 (1.0–2.1)	**<0.001**	1.2 (0.8–1.7)	1.2 (0.8–1.7)	1.2 (0.8–1.8)	**0.023**	**<0.001**
Smoking				0.094				**0.001**	**<0.001**
Never smoker	2072 (55.5)	238 (59.7)	1834 (55.0)		1626 (56.7)	996 (59.9)	630 (52.2)		
Current smoker	877 (23.5)	77 (19.3)	800 (24.0)		478 (16.7)	248 (14.9)	230 (19.0)		
Ex-smoker	782 (21.0)	84 (21.1)	698 (21.0)		766 (26.7)	418 (25.2)	348 (28.8)		
Low/middle education	2837 (76.0)	260 (65.2)	2577 (77.3)	**<0.001**	2181 (76.0)	1302 (78.3)	879 (72.8)	**0.001**	0.966
Low/lower middle equivalent income	1862 (49.9)	154 (38.6)	1708 (51.3)	**<0.001**	1474 (51.2)	922 (55.5)	549 (45.5)	**<0.001**	0.278
Macrovascular disease	160 (4.3)	15 (3.8)	145 (4.4)	0.581	106 (3.7)	58 (3.5)	48 (4.0)	0.498	0.223
Myocardial infarction	100 (2.7)	9 (2.3)	91 (2.7)	0.578	84 (2.9)	47 (2.8)	37 (3.1)	0.712	0.546
Stroke	69 (1.9)	6 (1.5)	63 (1.9)	0.587	26 (0.9)	14 (0.8)	12 (1.0)	0.673	**0.001**

Data are N (%), mean ± SD or median (25th; 75th percentile).

a *p*-values for differences between low/middle and high deprivation, significant differences (*p* < 0.05) are highlighted in bold.

b *p*-values for differences between SHIP-TREND and KORA-F4, significant differences (*p* < 0.05) are highlighted in bold.

### Relationship of Individual Socioeconomic Status and Area Deprivation With Macrovascular Disease

Table 2 summarizes the results of univariate and multivariate logistic regression analyses, in which the association of macrovascular disease with individual socioeconomic status (educational level, equivalent income) and area deprivation (GIMD) was examined. In crude models, participants with low/middle education had significantly increased odds of having macrovascular complications compared to persons with high education (OR = 1.58, 95% CI 1.14–2.18). In male participants with low/middle educational level, the OR was 1.84 (95% CI: 1.26–2.69) compared to men with high educational level. In women, educational level was not related to macrovascular disease. Area deprivation as well as equivalent income were not associated with myocardial infarction or stroke in any of the crude models.

**TABLE 2:** Association between macrovascular disease, educational level, equivalent income and area deprivation in study of health in Pomerania (2008–2012) and cooperative health research in the region Augsburg (2006–2008); results of regression analyses, adjusted for different covariate sets, Germany.

Variable	Macrovascular disease (yes vs. no)		
	Total	Men	Women
	OR (95% Cl**)**	OR (95% Cl)	OR (95% Cl)
**Crude models**			
SHIP-TREND vs. KORA-F4	1.17 (0.91–1.50)	1.31 (1.00–1.76)	0.92 (0.57–1.46)
Men vs. women	**2.92 (2.22–3.84)**	–	–
Age (per year)	**1.10 (1.09–1.12)**	**1.10 (1.09–1.12)**	**1.09 (1.07–1.12)**
Low/middle vs. high education	**1.58 (1.14–2.18)**	**1.84 (1.26–2.69)**	1.47 (0.77–2.81)
Equivalent income		
upper middle (vs. high)	1.25 (0.85–1.83)	1.21 (0.78–1.89)	1.72 (0.78–3.82)
lower middle (vs. high)	1.30 (0.89–1.89)	1.41 (0.91–2.16)	1.51 (0.68–3.34)
low (vs. high)	1.20 (0.77–1.86)	1.38 (0.83–2.28)	1.25 (0.50–3.14)
GIMD (low vs. high deprivation)	1.21 (0.92–1.59)	1.10 (0.80–1.51)	1.54 (0.89–2.66)
**Models 1–5** **^a^**			
1. SHIP-TREND vs. KORA-F4	**1.37 (1.06–1.78)**	**1.54 (1.13–2.11)**	1.04 (0.65–1.67)
Low/middle vs. high education	**1.46 (1.04–2.05)**	**1.72 (1.16–2.55)**	0.85 (0.44–1.65)
2. SHIP-TREND vs. KORA-F4	**1.44 (1.10–1.89)**	**1.63 (1.17–2.26)**	1.08 (0.66–1.76)
Equivalent income		
upper middle (vs. high)	1.20 (0.80–1.79)	1.14 (0.72–1.82)	1.38 (0.61–3.12)
lower middle (vs. high)	1.21 (0.82–1.79)	1.17 (0.75–1.83)	1.36 (0.61–3.03)
low (vs. high)	**1.60 (1.01–2.56)**	**1.74 (1.01–3.00)**	1.35 (0.52–3.50)
3. SHIP-TREND vs. KORA-F4	**1.36 (1.01–1.85)**	**1.67 (1.15–2.41)**	0.87 (0.51–1.49)
GIMD (low vs. high deprivation)	0.98 (0.70–1.36)	0.81 (0.54–1.20)	1.53 (0.82–2.86)
4. SHIP-TREND vs. KORA-F4	**1.39 (1.02–1.89)**	**1.72 (1.18–2.51)**	0.87 (0.51–1.48)
Low/middle vs. high education	**1.46 (1.04–2.05)**	**1.72 (1.16–2.55)**	0.84 (0.43–1.64)
GIMD (low vs. high deprivation)	0.97 (0.69–1.36)	0.80 (0.54–1.19)	1.53 (0.82–2.86)
5. SHIP-TREND vs. KORA-F4	**1.45 (1.11–1.91)**	**1.66 (1.20–2.31)**	1.07 (0.65–1.75)
Low/middle vs. high education	1.40 (0.98–2.00)	**1.68 (1.11–2.53)**	0.77 (0.38–1.55)
Equivalent income		
upper middle (vs. High)	1.14 (0.76–1.71)	1.07 (0.67–1.71)	1.46 (0.64–3.34)
lower middle (vs. high)	1.09 (0.73–1.63)	0.99 (0.62–1.57)	1.47 (0.64–3.38)
low (vs. high)	1.44 (0.89–2.32)	1.47 (0.84–2.57)	1.46 (0.55–3.91)
**Models 6–10** **^b^**			
6. SHIP-TREND vs. KORA-F4	1.17 (0.88–1.55)	1.26 (0.89–1.79)	0.88 (0.53–1.46)
Low/middle high education	1.39 (0.98–1.99)	**1.69 (1.12–2.56)**	0.69 (0.35–1.37)
7. SHIP-TREND vs. KORA-F4	1.22 (0.91–1.64)	1.33 (0.93–1.91)	0.92 (0.55–1.55)
Equivalent income		
upper middle (vs. high)	1.13 (0.75–1.71)	1.07 (0.66–1.74)	1.24 (0.54–2.84)
lower middle (vs. high)	1.15 (0.77–1.72)	1.09 (0.68–1.73)	1.21 (0.53–2.75)
low (vs. high)	1.54 (0.95–2.51)	1.66 (0.94–2.93)	1.24 (0.47–3.28)
8. SHIP-TREND vs. KORA-F4	1.18 (0.85–1.64)	1.39 (0.93–2.09)	0.77 (0.44–1.36)
GIMD (low vs. high deprivation)	0.94 (0.67–1.33)	0.78 (0.52–1.19)	1.43 (0.75–2.70)
9. SHIP-TREND vs. KORA-F4	1.20 (0.86–1.66)	1.42 (0.94–2.14)	0.76 (0.43–1.33)
Low/middle vs. high education	1.39 (0.98–1.99)	**1.68 (1.11–2.55)**	0.68 (0.34–1.36)
GIMD (low vs. high deprivation)	0.95 (0.67–1.34)	0.79 (0.52–1.20)	1.43 (0.76–2.70)
10. SHIP-TREND vs. KORA-F4	1.23 (0.92–1.65)	1.35 (0.94–1.94)	0.91 (0.54–1.53)
Low/middle vs. high education	1.34 (0.93–1.94)	**1.66 (1.08–2.54)**	0.64 (0.32–1.31)
Equivalent income		
upper middle (vs. high)	1.09 (0.72–1.65)	1.01 (0.62–1.65)	1.35 (0.58–3.12)
lower middle (vs. high)	1.06 (0.70–1.61)	0.94 (0.58–1.53)	1.35 (0.58–3.14)
low (vs. high)	1.41 (0.86–2.32)	1.41 (0.79–2.53)	1.40 (0.52–3.78)

OR, odds ratio; CI, confidence interval; GIMD, German Index of Multiple Deprivation.

a Models 1–5: adjusted for sex and age.

b Models 6–10: adjusted for sex, age and potential confounders (diabetes, BMI, blood pressure, LDL-cholesterol, triglycerides and smoking). Significant differences are highlighted in bold.

Fitting multivariable logistic regression models 1–5, adjusted for cohort, sex, age and educational level (Model 1), area deprivation (Model 2), equivalent income (Model 3), educational level and area deprivation (Model 4) and finally educational level and equivalent income (Model 5), we found that low/middle educational level in participants (total population and men) was significantly associated with an increased odds of having macrovascular disease. In both models 1 and 4, the OR was 1.46 (95% CI 1.04–2.05) for the general population and 1.72 (95% CI 1.16–2.55) for male participants. In model 5, male participants with low/middle educational level also had a significantly higher odds for having myocardial infarction or stroke than higher educated men (OR = 1.68, 95% CI 1.11–2.53). In all these models, area deprivation was not associated with macrovascular complications. Equivalent income was associated with macrovascular disease only in one model adjusted for cohort, sex, age and further potential confounders (Model 3). This model showed that both participants from the general population and men with low equivalent income had a higher odds of macrovascular complications compared to persons with high equivalent income (OR = 1.60, 95% CI 1.01–2.56 and OR = 1.74, 95% CI 1.01–3.00, respectively).

Models 6–10 were additionally adjusted for diabetes, BMI, blood pressure, LDL-cholesterol, triglycerides, and smoking. Low/middle education was significantly associated with myocardial infarction and stroke in men (OR = 1.69, 95% CI 1.12. – 2.56). This association was not changed (OR = 1.68, 95% CI 1.11–2.55) after further adjusting for area deprivation and educational level. Finally, the relationship persisted (OR = 1.66, 95% CI 1.08–2.54) after including both educational level and equivalent income. Equivalent income and area deprivation still had no impact on having macrovascular disease.

In all multivariable logistic regression models, men had a significantly increased odds (*p* < 0.001) of having myocardial infarction or stroke compared to female participants.

The logistic regression models, supplemented by interaction terms of GIMD and educational level (model 6) and GIMD and equivalent income (model 7) showed only a weak significance (*p* = 0.023) for men in Model 6.

## Discussion

For the first time in Germany, the impact of three different socioeconomic determinants on macrovascular outcomes using data from two large population-based surveys were analyzed. The data showed a higher odds of prevalent myocardial infarction and stroke in men with low and middle educational level compared to men with high education. Further analyses revealed that the association remained unchanged after further adjusting for potential confounders (diabetes, BMI, blood pressure, LDL-cholesterol, triglycerides and smoking). In women, we found no association of educational level and macrovascular disease. Finally, neither individual equivalent income nor area deprivation was related to macrovascular complications in both sexes. The additional analysis showed no considerable interaction between area deprivation and individual socioeconomic characteristics on the prevalence of macrovascular disease.

In the present analysis, education was inversely associated with macrovascular disease, which has been described in some but not all previous studies [4, 9, 10, 18, 19]. For example, in a Mendelian randomization [4] that included 543,733 participants and used genetic variants associated with education, low education was a causal risk factor of coronary heart disease. It was estimated, that 3.6 years of additional education reduce the risk of coronary heart disease by about one-third. A systematic review and meta-analysis, that examined 72 cohorts from Europe, United States and Asia [9] concluded that participants with low education have an increased risk of coronary artery disease (RR = 1.36, 95% CI 1.11–1.66), cardiovascular events (RR = 1.50, 95% CI 1.17–1.92), stroke (RR = 1.23, 95% CI 1.06–1.43) and cardiovascular death (RR = 1.39, 95% CI 1.26–1.54) compared to participants with higher education. A further study from France with 19,808 participants [10], which analyzed the relationships between socioeconomic characteristics and coronary heart disease revealed results similar to our study. In a model, conducted in men (due to the small number of cases in women), the hazard ratio for coronary heart disease in men with low educational attainment was 1.61 (95% CI 1.01–2.55) compared to men with high educational attainment. Moreover, the authors reported that coronary heart disease incidence was not associated with household income. Additionally, the coronary heart disease risk difference between municipalities with a low and high social status was not statistically significant after controlling for risk factors.

The associations between area deprivation and individual socioeconomic status with cardiovascular disease or cardiometabolic risk factors have also been investigated in a number of studies [6, 10, 20–23]. In a systematic review by Toms et al. [6], 24 studies investigating geographic and area-level socioeconomic variation in cardiometabolic risk factor distribution reported associations of higher prevalence of hyperglycaemia, dyslipidaemia, BMI, blood pressure and reduced glomerular filtration rate with greater area-level socioeconomic disadvantage, which is in contrast to our results. The associations were independent of individual-level characteristics such as income, education and occupation. A further study in Sweden [20], conducted with more than one million participants between 40 and 50 years, showed a higher risk of both myocardial infarction and coronary heart disease in individuals living in neighborhoods with low socioeconomic status. Moreover, an analysis which included 256,466 Indians and Europeans aged 30–74 years in New Zealand [22] also indicated a linear association of quintiles of socioeconomic deprivation with cardiovascular disease in both ethnic groups. The present study adds to this evidence that in Germany, the largest population in Europe, a more differentiated association of socioeconomic factors with macrovascular disease exists. Low education was the solely relevant risk factor in men only.

The presence of a higher odds for myocardial infarction and stroke in persons with lower educational level may be attributed both to direct or indirect factors. First, as shown by Mackenbach et al., persons with lower educational level were more often current cigarette smokers, less often moderate alcohol consumer, had more often overweight and consumed less frequently fresh vegetables [24]. Moreover, they did not participate in vigorous or moderate physical activity [25]. These are all well-known risk factors for macrovascular disease (2009). Furthermore, people in lower education groups frequently had poor health literacy, which supposes a reduced ability to understand comprehend medical information and poor adherence of medication [26]. In addition, there is evidence that the propensity to use preventive health services, such as regular medical check-ups, was less frequent among persons with low level of education [27]. Finally, people in lower education groups lived more often in more air-polluted areas [28], which was also identified as a risk factor for myocardial infarction, stroke and heart failure [29]. Overall, the mortality from cardiovascular disease was higher among men and women with a lower than with a higher education [30].

The present study has a number of strengths. First, it is based on two large population-based samples of well-characterized participants, which is a unique resource for studying the macrovascular outcomes. Second, it includes detailed and highly comparable data on socioeconomic variables, area deprivation, lifestyle and multiple risk factors. Lastly, including two individual socioeconomic variables as well as an area-based deprivation measure, we were able to simultaneously evaluate the impact of different social determinates on the prevalence of macrovascular disease.

However, the study has also some limitations. Firstly, it is possible that the prevalence of macrovascular disease was underestimated in the South of Germany since in the follow-up survey (KORA-F4) persons with higher morbidity most likely had a higher likelihood of not attending the follow-up investigation. Secondly, the information on the household income in our study was self-reported and therefore limited by a respondent’s lack of willingness to reveal it. Therefore, all our models were based on complete case analysis. Thirdly, the data on myocardial infarction and stroke were also self-reported. However, the self-reported data for these acute events generally show good validity [31]. Furthermore, information on traditional risk factors such as excessive alcohol drinking or physical inactivity were not included in the analysis. This was due to different assessment methods of alcohol consumption in SHIP-TREND and KORA-F4 and imprecize assessment of the physical activity level. In the KORA study, the population in the lower deprived area had a higher percentage of low educated people than in the higher deprived area. Most likely, this was due to the fact that the KORA study has on average older participants compared to the SHIP study. In the older KORA population, the proportion of participants with low and medium education is relatively high. Until well into the 1970/1980s, this was a standard among the general population (around 70%). The proportion of persons with lower secondary school qualifications later decreased in younger age groups.

Moreover, due to privacy regulations we were not able to use multilevel models to take into account individual and area level effects. Finally, it must be noted that the present study is of cross-sectional design so that cause and effect relationships cannot be stated.

### Conclusion

In conclusion, the results of our study suggest that only individual educational level plays a role in the explanation of the prevalence of macrovascular disease in males. Neither individual equivalent income nor area deprivation are associated with macrovascular complications in both sexes. The present study suggests that higher education may lead to cardiovascular health benefits. Nevertheless, further research is needed to gain a better understanding of causal pathways of education on macrovascular outcomes and to fulfill the research needs in this field. Such findings would be a major step forward for effective preventive health and education policies.

## Data Availability

The data analyzed in this study is subject to the following licenses/restrictions: Data are subject to national data protection laws and only available upon formal request. Requests to access these datasets should be directed to for KORA-F4: AP (peters@helmholtz-muenchen.de), for SHIP-TREND: https://www.fvcm.med.uni-greifswald.de/dd_service/data_use_intro.php
